# Homology and Architecture of the Caudal Basket of Pachycephalosauria (Dinosauria: Ornithischia): The First Occurrence of Myorhabdoi in Tetrapoda

**DOI:** 10.1371/journal.pone.0030212

**Published:** 2012-01-17

**Authors:** Caleb Marshall Brown, Anthony P. Russell

**Affiliations:** 1 Department of Ecology and Evolutionary Biology, University of Toronto, Toronto, Ontario, Canada; 2 Department of Biological Sciences, University of Calgary, Calgary, Alberta, Canada; Raymond M. Alf Museum of Paleontology, United States of America

## Abstract

**Background:**

Associated postcranial skeletons of pachycephalosaurids, most notably those of *Stegoceras* and *Homalocephale*, reveal enigmatic osseous structures not present in other tetrapod clades. The homology and functional significance of these structures have remained elusive as they were originally interpreted to be abdominal ribs or gastralia, and more recently have been interpreted as *de novo* structures in the tail.

**Principal Findings:**

Analysis of these structures in nearly all pachycephalosaurid skeletons has facilitated a complete description of their architecture, and the establishment of patterns consistent with those of myorhabdoid ossifications — ossifications of the myoseptal tendons associated with myomeres. The presence and structure of myorhabdoid ossifications are well established for teleost fish, but this marks their first recognition within Tetrapoda. These elements are both structurally and histologically distinct from the deep, paraxial ossified tendon bundles of other ornithischian clades, although they may have performed a similar function in the stiffening of the tail.

**Conclusions/Significance:**

These myorhabdoi are not *de novo* structures, but are instead ossifications (and therefore more amenable to fossilization) of the normally unossified plesiomorphic caudal myosepta of vertebrates. The ubiquitous ossification of these structures in pachycephalosaurids (all specimens preserving the tail also exhibit myorhabdoid ossifications) suggests it is a likely synapomorphic condition for Pachycephalosauria.

## Introduction

**Figure 1:**
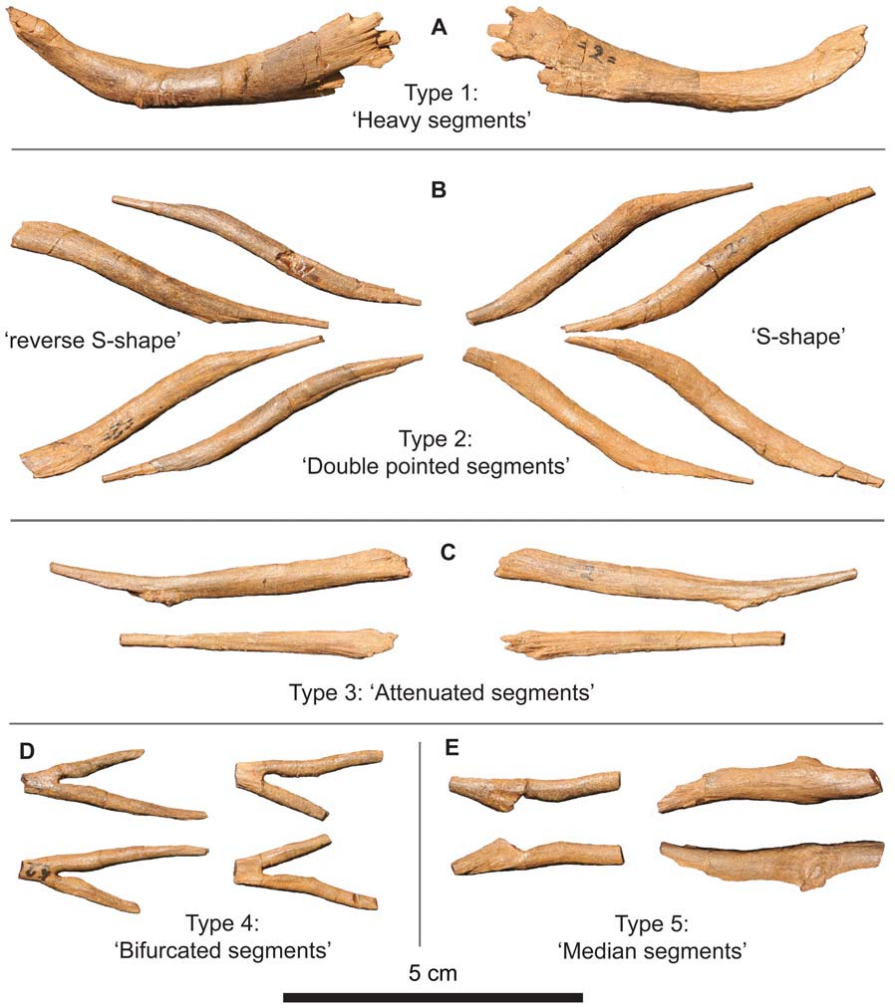
Elements typifying the five tendon morphotypes of Gilmore [**3**] in internal and external views. **A**) Type 1 – ‘Heavy segments’ – “Large, heavy, sinuous segments with more or less flattened ends that are sometimes grooved with ray-like points” ([3]:31). These show right and left symmetry. One element reflected in the vertical plane: left; external view, right; internal view. **B**) Type 2 – ‘Double pointed segments’ – “Smaller, subround, sinuous segments having both ends slightly flattened and slenderly pointed” ([3]:32). Two elements of each ‘side’ are shown, reflected in the horizontal plane: upper; external view, lower; internal view. **C**) Type 3 – ‘Attenuated segments’ – “Of about the same size as the second, subround, sinuous, with one flattened end, the other drawn out into a slender attenuated rod-like process” ([3]:32). Two elements reflected in the vertical plane and of uncertain orientation. **D**) Type 4 – ‘Bifurcated segments - Rare (only two elements known). “Small, with long bifurcated divergent rounded processes at one end, with the other end unknown” ([3]:32). Two elements reflected in the horizontal plane: upper; external view, lower; internal view. **E**) Type 5 – ‘Median segments’ - Also rare. “Three small bones that are suggestive of being bilateral median segments, but the fact that both are asymmetrical would rather negative this suggestion” ([3]:32). These were incorrectly labeled as Type 4 four in Plate 14 of [3]. Two elements reflected in the horizontal plane and of uncertain orientation.

The dome-headed pachycephalosaurids remain the most enigmatic and poorly understood clade of ornithischian dinosaurs [1]. Upon its discovery in 1921, and its description in 1924, UALVP 002 (*Stegoceras validum* Lambe) was the first pachycephalosaurid dinosaur for which significant postcranial skeletal material was known, and revealed much about the anatomy of this clade [2]–[4]. Among the preserved material were dozens of small, disarticulated, bony elements that presented morphologies inconsistent with known anatomical structures of ornithischians (Figure 1). These were tentatively identified as “abdominal ribs” (gastralia) or possibly “ossified tendons” by Gilmore [3]. The presence of gastralia, if verifiable, would be unique within the Ornithischia. These elements were categorized by Gilmore into five morphotypes, some of which showed what appeared to be bilateral symmetry (Figure 1).

**Figure 2:**
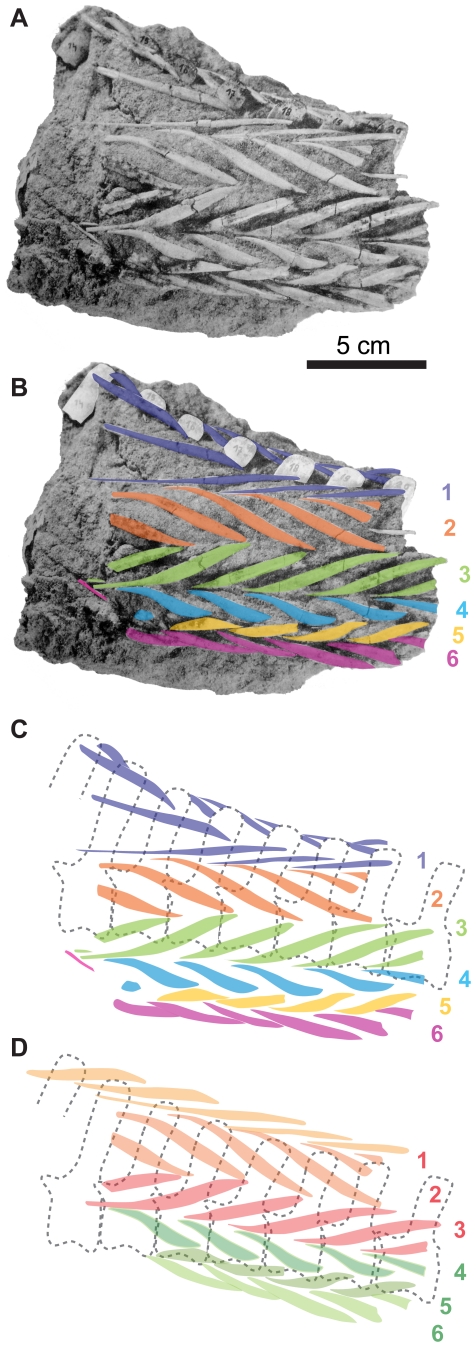
Articulated caudal tendons of *Homalocephale* in lateral view. Articulated caudal tendons in matrix block from the 14th to 20th caudal vertebrae of *Homalocephale* (MPC-D 100/1201) in left lateral view. **A**) photograph of articulated block without interpretation (modified from Maryańska and Osmólska [5]). **B**) colored overlays of putatively serially homologous tendinous elements illustrating the six rows noted by Maryańska and Osmólska [5]. **C**) Matrix removed showing position of tendon rows relative to the underlying vertebrae. **D**) Reconstructed tendons after elimination of the ventral slumping and slight rotation of the articulated tendons relative to the underlying vertebrae.

Subsequent discovery of an articulated set of “caudal tendons” in a portion of the tail of *Homalocephale calathocercos* Maryańska and Osmólska (Figure 2A) (MPC-D 100/1201 – formerly GI SPS 100/51) [5], [6] led to the establishment of homology between the “abdominal ribs” of Gilmore [3] and the caudal “basket-work of tendons” of Maryańska and Osmólska [5] and led to the rejection of their interpretation as gastralia in *Stegoceras* [7]. Similar disarticulated structures were also noted for *Prenocephale prenes* Maryańska and Osmólska (Z. Pal. No. MgD-I/104; [5]) and *Goyocephale lattimorei* Perle, Maryańska and Osmólska (MPC-D 100/1501 – formerly GI SPS 100/1501; [8]). These articulated elements were described as an intricate “basket” consisting of six parallel rows of fusiform “tendons” with the attenuated extremities of each making contact with the elements of the adjacent row.

Sues and Galton [9] attempted to assign the five “abdominal rib” types of Gilmore [3] to the serially homologous tendon rows of the caudal “basket” of *Homalocephal*e [5]. They interpreted the Type 1 elements (Figure 1A) as representing the thickened medial portions of the elements of the fourth and fifth row of the caudal “basket” (Figure 2A, 2B); Type 2 elements (Figure 1B) were stated to be similar to the medial portions of the elements from the second and third rows (Figure 2A, 2B); and Type 3 elements (Figure 1C) were stated to represent the posterior portions of the elements represented as Type 2 (Figure 1). Type 4 elements (Figure 1D) were thought to be the products of fusion between the rod-like posterior portions of caudal structures, but could not be matched with any elements of the caudal “basket” in *Homalocephale* (Figure 2A). Sues and Galton were also unable to confidently match Type 5 elements (Figure 1E) with any known structures. This attempt [9] to match the isolated elements found associated with *Stegoceras validum* with the preserved *in situ* structures of *Homalocephale* resulted in apparent partial success, but confusion was introduced because of a reversal in labeling. In the figures presented by Sues and Galton [9] labels 1, 2, 3, 4 and 5 actually refer to types 4, 5, 3, 2, and 1 respectively, as originally indicated by Gilmore [3] (Figure 1).

Overall, the “ossified tendons” described by Gilmore [3] in *Stegoceras* were interpreted, by Sues and Galton [9], to be elements of the “caudal basket” from a region more distal in the tail than the region preserved in *Homalocephale* (MPC-D 100/1201 – formerly GI SPS 100/51). This potentially accounted for the imperfect match between the examples preserved in these two taxa. Sues and Galton [9], however, concluded that the “caudal basket” indicated that the tail of pachycephalosaurs was highly specialized, and they thus endorsed the postulation of Maryańska and Osmólska [5] that it acted as one component of the “tripodal prop”, along with the two hind limbs.

Goodwin et al. [10] subsequently reported the occurrence of “ossified tendons” in a fourth taxon of pachycephalosaurid, *Stygimoloch spinifer* Galton and Sues. UCMP 128383 preserves isolated fragments of Gilmore's Type 1 and 3 elements (Figure 1A and C), and AMNH 21541 preserves isolated fragments of unspecified type. Goodwin et al. suggested that the “ossified tendons” in the caudal region may have served a protective function by acting as a cuirass against agonistic behaviors such as flank-butting. The isolated “ossified tendons” of *Stygimoloch* were sectioned by Organ and Adams [11] and found to be similar, histologically, to other ornithischian tendons in their internal architecture, except for the unique possession of alternating longitudinal and radial vascularization, contrasting with the longitudinal pattern seen in all other dinosaur clades.

Although these caudal elements have generally been accurately described, both from isolated structures of *Stegoceras* (UALVP 002; [3]) and articulated elements *in situ* in the tail of *Homalocephal*e (MPC-D 100/1201; [5]), and the identity between the two established [5], [9], their anatomical relationships have not been thoroughly explored. Unnecessary assumptions have, therefore, resulted in the postulation that these elements comprise a *de novo* autapomorphic “caudal basket” in the Pachycephalosauridae.

### Institutional abbreviations

**AMNH**, American Museum of Natural History, New York, New York, USA; **MPC**, Paleontological Center, Mongolian Academy of Sciences (formerly Section of Paleontology and Stratigraphy of the Geological Institute, Mongolian Academy of Sciences), Ulaanbaatar, Mongolia; **NHMUK**, Natural History Museum (formerly British Museum of Natural History), London, UK; **UALVP**, University of Alberta Laboratory of Vertebrate Paleontology, Edmonton, Alberta, Canada; **UCMP**, University of California Museum of Paleontology, Berkeley, California, USA; **Z. Pal.**, Palaeozoological Institute of the Polish Academy of Science, Warsaw, Poland.

## Materials and Methods

Here we report on nearly all significantly complete pachycephalosaur postcranial skeletons that preserve portions of the tail and that are housed in accessible institutions. These are as follows: *Homalocephale calathocercos* (MPC-D 100/1201 — formerly GI SPS 100/51); *Stegoceras validum* (UALVP 002); *Prenocephale sp.* (MPC-D 100/1204); and *Stygimoloch spinifer* (UCMP 128383 and AMNH 21541).

*Homalocephale calathocercos* (MPC-D 100/1201) reveals the most extensive set of articulated *in situ* ossified elements, and their articulation provides the most robust evidence for the interpretation of their anatomical identity and their broader-scale homology. Both *Stegoceras validum* (UALVP 002) and *Prenocephale sp.* (MPC-D 100/1204) exhibit extensive, but less well-preserved, isolated elements, as well as smaller blocks of articulated or *in situ* elements. *Stygimoloch spinifer* (UCMP 128383) preserves large and robust isolated elements, but no articulated sets.

Photographs were taken using a 10.1 MP Canon EOS 40D with a Canon macro lens. Alterations to photographs (e.g. removal of backgrounds) were performed using Adobe Photoshop 10.0.0. Drawings were created in Adobe Illustrator 13.0.0. Measurements were taken using 15 cm digital calipers.

## Results

### Description of the elements

The pachycephalosaur caudal elements in question have previously been described in both their isolated [3] and articulated [5] states. Those descriptions are accurate in terms of the general form and pattern reported on. The following description serves to supplement these.

#### *Homalocephale calathocercos* (MPC-D 100/1201)

The holotype of *Homalocephale calathocercos* (MPC-D 100/1201 - formerly G.I. No. SPS 100/51) exhibits an articulated set of caudal elements on the left side of the tail, spanning from the 14th to the 20th caudal vertebra (Figure 2A). These are preserved *in situ*, within a matrix block that encases the caudal vertebrae. The right side of the block also exhibits a smaller number of elements, which, are largely shifted from their original positions and associations. A second smaller, mostly disarticulated but *in situ*, congregation of elements is preserved spanning the 24th to the 26th caudals, also on the left side of the tail.

**Figure 3:**
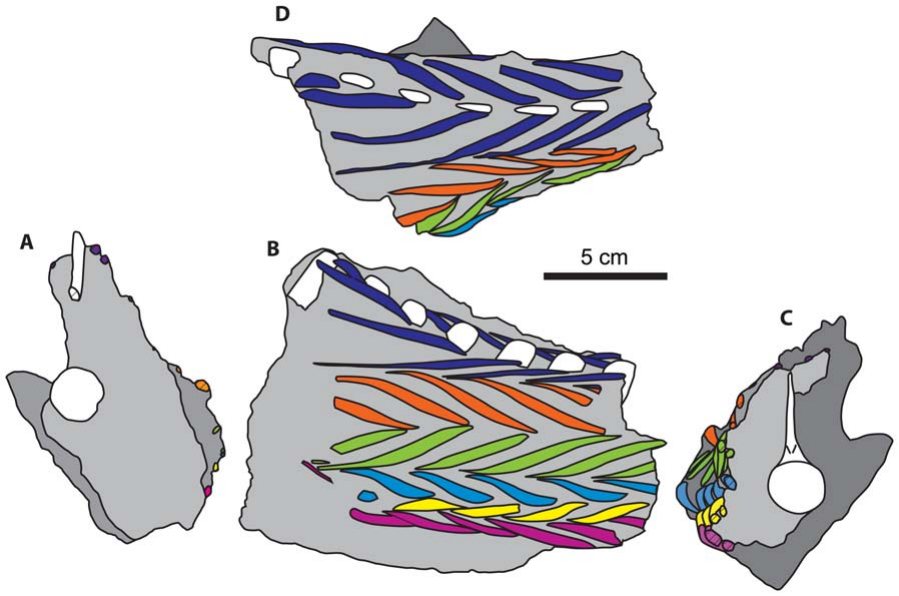
Schematic of the articulated caudal tendons of *Homalocephale* in multiple views. A schematic diagram of the articulated caudal tendons in the matrix block from the 14th to 20th caudal vertebrae of *Homalocephale* (MPC-D 100/1201) in **A**) anterior, **B**) left lateral, **C**) posterior, and **D**) dorsal views. White = bone, grey = matrix, and colors = tendons rows.

The articulated elements of the anterior block display a distinctive and consistent arrangement along the length of their preserved occurrence. Six rows of elements are evident, and these are arrayed in parallel with each other (Figure 2A, 2B). The dorsalmost row is positioned just lateral to the neural spines, and the ventralmost is positioned just lateral to the ventral tips of the chevrons. The four intervening rows arch laterally between these (Figures 2B, 3). Following the convention of Maryańska and Osmólska [5] we number these rows from dorsal to ventral (1–6) (Figure 2B, 2C).

The ossified elements within each row are fusiform and sigmoidal, with thickened middle portions and curving, attenuated extremities. The elements of each row are positioned with the long axes of the thickened middle sections diagonal to the frontal plane (and long axis of the row). The deflection from the frontal plane alternates in direction between adjacent rows (Figure 2A, 2B), resulting in a ‘zig-zag’ pattern (Figure 2A, 2B, 3B). The attenuated extremities of each element curve to ultimately lie adjacent to, and in parallel with, their counterparts in the adjacent rows. The elements are also slightly bowed in the transverse plane so that the thickened middle portions consistently lie more superficially (closer to the interface of the caudal musculature and dermis) than do their attenuated extremities, with the latter projecting deeper into the block, often becoming obscured by matrix or the overlapping element of the adjacent row.

The long axes of the elements of the first row (purple) trend anteroventrally in relation to the neural spine, such that they approach their contralateral counterpart at the dorsal midline (Figure 2B, 2C, 3B, 3D). They eventually disappear from view as they course anteroventrally, the ventral extremities being longer and more attenuated than the dorsal extremities, although this may be the result of the dorsal extremities being obscured in their most distal regions. The ventral extremities project anteriorly, converging with, and running parallel to, the anteriorly-projecting dorsal extremities of the second row (red) (Figure 2B, 2C, 3B, 3D). The convergent anterior projections of adjacent elements in rows one and two extend deep to, and are obscured by, the convergent anterior projections of the next more anterior pair, which are in turn overlain by the next more anterior pair. The long axes of the thickened middle portions of the elements of the second row trend posteroventrally and form angles of between 31° and 34° with those of the first row. Elements of the second row (red) are slightly thicker than those of the first and form angles of between 47° and 58° with the adjacent elements of the third row (green) (Figure 2B, 2C, 3B). As is the case for the junctions between rows one and two, those for rows two and three converge upon each other (Figure 2B, 2C). Following this convergence, the posteriorly-directed extremities course medially and are obscured by matrix.

The elements of the third row (green) are almost perfect mirror images of those of the second row (red), and their ventral extremities project anteriorly, taper, and converge with the tapering anteriorly-projecting dorsal extremities of the fourth row (blue) (Figure 2B, 2C). The angle between the major axes of the elements of rows three and four is between 30° and 45°. The ventral extremities of the elements in row four (blue) project posteriorly, as do the dorsal extremities of the fifth row (yellow), but the convergence of these two is not discernible because of their highly arched nature (Figure 2B, 2C). The angles between the long axes of the elements of the fourth and fifth rows range from 20° to 28°. The anteriorly-projecting ventral extremes of the elements in the fifth row (yellow) converge with those of the sixth (magenta), the latter being located entirely ventrally, with their posteriorly-projecting medial extremities running parallel to the ventral midline, as indicated by the ventralmost extremities of the chevrons.

In addition to the elements preserved on the left side, the right side of the block preserves much of row one, which is a mirror image (relative to the dorsal midline) of that of row one of the left side (Figure 3D). The main axes of the left and right first rows lie at angles of between 35° and 45° to each other, with the left lying at 20°–25°, and the right at 14°–20° to the dorsal midline. The majority of the remaining rows of the right side are missing, with only a few disarticulated and randomly associated fragments preserved.

Whereas the pattern of orientation and articulation of the ossified elements is consistent across the rows, there are some distinctions between rows, most notably between the dorsal three rows (one, two, and three) and the ventral three rows (four, five, and six). The elements of the ventral three rows are distinctly more rounded in cross-section than are those of the dorsal three rows, which are more compressed and oval in cross-section. In addition to the difference in shape, the elements of the ventral rows are more highly bowed (resulting in a greater degree of arching out of the matrix), are spaced more closely together vertically (their anteroposterior spacing is consistent), and are oriented with their counterpart in the adjacent rows at lower angles (Figures 2, 3). Along the caudal vertebral series over which they are preserved, the pattern of architecture of the elements remains consistent, with a general tendency for both reduced thickness of the elements and reduced angle of orientation with respect to one another as the series are traced proceeding posteriorly.

Incomplete anterior and posterior margins of the preserved ossified complex, and broken edges of the matrix block collectively expose partial transverse sections of the tail, revealing some of its internal structure (Figure 3A, 3C). The externally visible extent of the elements accounts for only one half to one third of their entire length, and is dominated by the thickened and diagonally oriented middle portions that usually cross two or more vertebral segments (Figure 2C). The thinning extremities that are oriented more or less parallel to the frontal plane contribute only a small component before being lost from view. In instances where the overlying matrix has been removed, the length of the attenuated extremities can be followed. This is so for the anteriorly-projecting ventral extremities of the third from anterior element preserved in row one (Figures 2B, 3B), the anteriorly-projecting dorsal/lateral extremity of the anteriormost element preserved in row six on the left side (Figures 2B, 3B), and the anteriorly-projecting ventral extremity of the elements in row six on the right side (Figure 3B). In these cases the attenuated extremity of a single half of the element traverses up to three vertebral segments, suggesting that a complete element would span more than six vertebral segments. As noted by Maryańska and Osmólska [5], the converging extremities of adjacent rows are often fused together, forming a “V” like structure.

The semicircular arc of the superficial thickened portions of the elements does not lie adjacent to the vertebral column; rather, it is limited to the circumference of a cylinder of matrix that surrounds the caudal vertebrae (Figure 3A, 3C). Whereas the thickened middle portions of the tendons are superficial, their attenuated extremities course slightly deeper. The overlapping nature of these convergent extremities yields an overall form of a series of stacked Vs, that are largely superficial.

#### *Prenocephale sp.* (MPC-D P100/1204)

A large, recently-discovered specimen of *Prenocephale* (MPC-D P100/1204) includes several small matrix blocks that reveal several superficial *in situ* caudal elements, as well as numerous isolated, and often fragmentary, superficial caudal elements. One of these blocks incorporates a small portion of the articulated network from the distalmost region of the tail. The ‘zig-zag’ nature seen in the articulated block of *Homalocephale* is also evident here. The angle of about 20° between the adjacent elements, however is slightly smaller than that seen in the more proximal portion of the tail of *Homalocephale*. The posterior elements are minute, having minimum diameters of less than 1 mm, and form a complete halo around the distal caudal centra.

A small block of matrix containing a rib in articulation with the transverse process of a dorsal vertebra also reveals parasagittal ossified elements. These are located dorsolateral to the articulation of the rib and transverse process. Too few elements, however, are preserved to enable establishment of their orientation to one another.

In addition to the *in situ* elements, numerous isolated elements are preserved. The majority of these are fragmentary, but a few are nearly complete. The isolated elements are all accounted for by the morphotypes identified by Gilmore [3] and correspond with the morphology of the elements described above for *Homalocephale*. The vast majority of them are assignable to Type 2, with a small number of Type 1 structures. There are no identified occurrences of Types 3, 4 and 5. Not surprisingly, the majority of these tendons are much larger than those preserved in the distal portion of the tail, with the largest measuring 10 mm in diameter.

#### *Stegoceras validum* (UAVLP 002)

*Stegoceras validum* (UAVLP 002) preserves not only the greatest number and best-preserved isolated caudal elements, but also the greatest diversity of form (Figure 1). A comprehensive survey of these elements, using the anatomy of the articulated morphology of the elements of *Homalocephale* and *Prenocephale* as comparators, raises questions about some of the original interpretations. Only three distinct and discrete morphotypes, represented by multiple specimens, are recognizable, corresponding to types 1, 2 and 3 (sensu [3]). Types 4 and 5 are recognized herein as broken or fused (or both) portions of type 2 tendons.

Type 1 (Figure 1A) are the largest and most robust of the preserved elements. They are sigmoidal, with rounded or slightly oval middle portions (in cross section) and bear compressed, paddle like extremities that are often divided distally into numerous ray-like projections. Orthogonal to the plane of the sinuosity, there is a distinct bowing of the long axis. The convex side of the middle portion invariably bears rugose swellings that are oriented perpendicular to the long axis, whereas the surface of the concave side is characterized by many longitudinally-oriented striae that grade distally into the projecting rays. Type 2 (Figure 1B) are sigmoidal and fusiform, and are thinner than those assignable to type 1. Their rounded or slightly oval middle portions gradually attenuate, flatten and curve into a distinct S-shape. As with type 1, there is a bowing of the long axis. Type 2 elements lack the transverse rugosities of type 1, but show the longitudinal striae on the convex surface. Type 3 elements (Figure 1C) are very similar to type 2, differing only in that they are more compressed and less rounded in cross section, are less bowed, and consist of straighter segments with less of a sigmoid curve. They too show the longitudinal striations of the other types.

**Figure 4:**
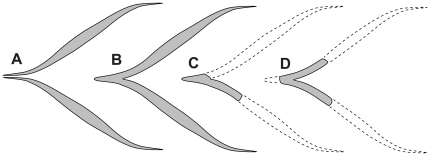
Fusion of adjacent tendon pairs. Unfused (**A**) and fused (**B**) type 2 tendons as seen in articulation in *Homalocephale* (MPC-D 100/1201) and *Stegoceras* (UALVP 002). Fused type 2 tendons with pattern of breakage resulting in type 5 (**C**) and type 4 (**D**) tendons seen in UALVP 002.

Types 4 and 5 (Figure 1D, 1E) are here determined not to represent unique morphotypes, but are rather able to be subsumed into type 2. As is evident from the articulated element sets of *Homalocephale* (MPC-D 100/1201), and as was observed by Maryańska and Osmólska [5], as the attenuated extremes of two adjacent rows converge they form a V-shape, and in many cases ‘fuse’ along their common axis of symmetry (Figure 4A, 4B). Fused pairs are also seen in the articulated matrix blocks of *Stegoceras* (UALVP 002) and as isolated elements in *Prenocephale* (MPC-D P100/1204). The bifurcated elements of the putative type 4 can be accounted for as the fused extremities of two adjacent type 2 elements segregated from their main bodies (Figure 4D). The same is true for one of the only two examples of putative type 5 (Figure 4C). The remaining example of type 5 of Gilmore [3] is asymmetrical, but does not share a common morphology with any other element.

**Figure 5:**
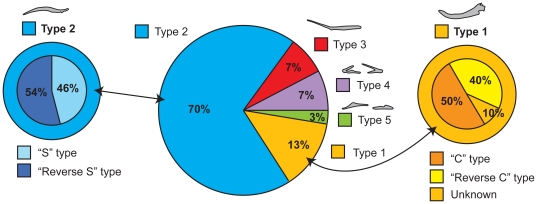
Pie chart showing relative proportions of representation of the caudal tendons of UALVP 002. Types 1 and 2 are further broken down showing the relative proportions of the mirror image conformations of each type.

Although types 4 and 5 were noted to be rare, Gilmore [3] and Sues and Galton [9] provided no data about the relative abundance of the other morphotypes of these caudal structures. The vast majority, 48 (70%), are assignable to type 2, only 9 (13%) are assignable to type 1, 5 (7%) are assignable to each of types 3 and 4, and only 2 (3%) are assignable to type 5 (Figure 5). If it is accepted that both types 4 and 5 are, in reality, truncated representatives of type 2, then the proportion of type 2 becomes 55/69 (80%).

Whereas it is possible to segregate the preserved elements of UALVP 002 into discrete morphotypes 1, 2 and 3, it is evident that a larger and more inclusive sample of elements may result in a breaking down of the distinction between such morphotypes. Instead one would have a continuous spectrum of morphology, without discrete types, as is evident in the articulated series of *Homalocephale* (Figure 2B, 2D). The discrete nature of the types is likely a result of the incomplete preservation of a continuum of expression of form.

Gilmore [3] discussed left/right symmetry in the sample of type 1 elements assigned to UALVP 002. Re-examination confirms the occurrence of left or right ‘handedness’ of both types 1 and 2, as evidenced by the bowing of the long axis. If the elements are laid on a flat surface so that the bowing (of their long axis) is positioned convex side up, type 2 tendons either exhibit an ‘S-shape’ (with the dorsal margin pointing right and the ventral margin pointing left) or a ‘reverse S-shape’ (a mirror image, with the dorsal extremity pointing left and the ventral extremity pointing right), and type 1 tendons either exhibit a ‘C-shape’ or a ‘reverse C-shape’. Both of these configurations are mirror-images of each other in three dimensions. The relative proportions of preserved left- and right-handed elements within each type are approximately equal, with type 1 being accounted for by four ‘C-shaped’, five ‘reverse C-shaped’ and one ambiguous unit, and type 2 being represented by twenty-two ‘S-shaped’ and twenty-six ‘reverse S-shaped’ elements (Figure 5).

**Figure 6:**
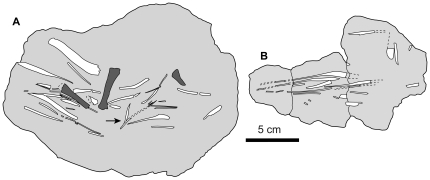
Illustrations of sandstone blocks with *in situ* caudal tendons of UALVP 002. **A**) A block showing the association of the dissociated elements and chevrons, reinforcing a caudal origin of the elements, and also illustrating the apical fusion of two paired elements in “V”: articulation (indicated by arrow). **B**) A smaller block from the more distal region of the tail illustrating the relative size of the thicker central portion of the elements versus the drawn-out and attenuated extremities, and the nearly parallel orientation of the attenuated extremities. Light grey = sandstone, white = caudal tendons, dark grey = chevrons.

In addition to the isolated elements, UALVP 002 also includes two small blocks of associated matrix containing *in situ* elements (Figure 6). The larger of these (Figure 6A) contains an association of the ossified elements and chevrons, as well as one pair of associated type 2 elements in the same “V” articulation as those of *Homalocephale* (MPC-D 100/1201). The smaller block (Figure 6B) contains a cluster of smaller ossified elements. Two of these elements show a distinct sigmoidal type 2 morphology, with their attenuated extremities being much thinner than the main body of the element, and running parallel to one another. These attenuated extremities exceed the preserved length of the main body of the elements and are morphologically equivalent to the medially-coursing (and almost entirely matrix-obscured) thinned portions of the elements in MPC-D 100/1201 (Figure 3B, 3D) (as noted by [5], [9]).

#### *Stygimoloch spinifer* (UCMP 128383)

Although no articulated elements are evident in this specimen, a large number of disarticulated and highly fragmented superficial caudal elements are preserved. These are much more robust than those seen in the other pachycephalosaurid specimens, having maximum widths and thicknesses of 16 mm and 13 mm respectively. Despite the fragmentary nature and incompleteness of these elements, their sigmoidal nature is apparent. The vast majority of these are assignable to the type 1 of Gilmore [3], although some of the smaller ones may be assignable to type 2. Their extremities are most often blunt, rather than tapered, with multiple ray-like processes projecting from the long axis of the element. This morphology is similar to that of type 1 elements of UALVP 002, although they are larger and more robust.

In addition to the superficial caudal elements, multiple caudal centra are preserved. These range in diameter from 41 to 28 mm, and, as such, represent the large anterior caudal vertebrae. There is no indication of the smaller, more distal caudals.

## Discussion

### Reconstruction

Examination of the articulated and isolated superficial caudal elements of the four pachycephalosaur specimens allows for a) a matching of the isolated elements of UALVP 002 with the articulated elements of MPC-D 100/1201, b) a revision of the types of elements (categories) present, and a documentation of their proportional representation, and c) a reconstruction of the three-dimensional architecture of the ossified caudal complex.

The association of the chevrons and superficial caudal ossified elements in the larger matrix block of UALVP 002 confirms the caudal origin of the isolated elements of UALVP 002 and further reinforces their identity with the articulated ones of MPC-D 100/1201. The morphology of type 3 elements is most similar to that of the first (dorsalmost) row of MPC-D 100/1201, with the flattened and straighter portions of the attenuated extremities lying adjacent to the neural spines and running parallel to the dorsal midline. This explains the asymmetry of the isolated type 3 elements, because the straight, attenuated posterior extremity is associated with neural spines. Type 2 elements match the morphology of rows two to six, expressing attenuated and sigmoidal extremities at both ends. Type 1 elements do not match any of those found on the articulated series of MPC-D 100/1201 and, as such, may originate from anatomical regions beyond the area preserved in the matrix block. Because these elements are thicker and more robust than those preserved in the articulated block, it is likely that they originated from a more proximal position, near the base of the tail. This is reinforced by the association of a preponderance of large and robust type 1 elements in UCMP 128383 with multiple anterior caudal centra and a noticeable lack of distal caudal centra, suggesting that only the anterior portion of the tail and its associated tendons were preserved.

The association of type 3 elements with the articulated row one units, and type 2 tendons with the articulated elements of rows two through six, leads to the prediction that a highly disproportionate representation of type 2 elements relative to type 3 elements should exist, in a ratio of approximately 5∶1. The ratio of the preserved type 2 and type 3 tendons seen in UALVP 002 should be similarly disproportionate. Indeed a ratio of 11∶1 is revealed.

Reconstruction of the structure of the complete superficial ossified caudal complex, based on extrapolation of the completely preserved left side and partially preserved right side of MPC-D 100/1201, the distal extremity of the tail of MPC-D P100/1204, and the isolated elements from UALVP 002, MPC-D P100/1204, and UCMP 128383, suggests a circular or oval halo of elements surrounding the entire caudal region. This halo is composed of six parallel rows forming a continuous arc on each side of the tail. Each respective row is composed of diagonally oriented sigmoidal elements. Elements of the dorsalmost row are oriented anteroventrally (beginning at the neural spine against which the posterior extremity of this element lies), with the direction alternating between adjacent rows resulting in the distinctive ‘zig-zag’ shape detailed by all series in combination (Figure 2A). Disposition of these elements about the transverse plane reveals that for the majority of the length of each they are restricted to the periphery of the oval. Only their anterior and posterior distal extremities project medially, and these do not deviate far from the surface. The cross sectional shape of the peripheral halo is elliptical, trending laterally from the dorsal tips of the neural spines, curving ventrally and approaching their maximum lateral extent at the location of the neurocentral suture, then curving medially to meet the ventral extremities of the chevrons in the ventral midline.

### Myoseptal morphology in craniates and homology of the pachycephalosaurid ossified caudal “basket”

The regularity, position and spacing of the caudal structures is suggestive that they are related to the segmental architecture of the tail. Indeed, although individual skeletal elements of this series span up to six vertebral segments, they serially repeat as a set of elements in such a way as to mirror the underlying vertebrae on a 1∶1 basis (Figure 2C, 2D).

Patterson and Johnson [12] conducted an extensive survey of intermuscular bones in teleost fishes and revealed that such elements can be expressed throughout the entire postcranial length of the body. Intermuscular bones are related to segmental muscle blocks, the myotomes, and the myoseptal complex that intervenes between them. Deep-seated intermuscular bones are normally associated with ligaments, and three series of these may be present — epineurals that are primitively associated with neural arches, epicentrals and epipleurals. The epineurals lie above the horizontal skeletogenous septum and have a posterodorsal to anteroventral orientation. The epicentral elements are associated with rib heads and lie in the horizontal septum. The epipleural elements lie below the horizontal septum and have an anterodorsal to posteroventral orientation. Patterson and Johnson [12] noted that the intermuscular bones are serially homologous segmental ossifications that are located in the myosepta. These ossifications have attachments to the axial skeleton.

Sporadically distributed among teleosts are other intermuscular bones that exist within the anterior and posterior flexures of the myosepta and that do not attach to the axial skeleton. These myorhabdoi [12], [13] are adventitious structures that occur superficially, just deep to the integument. As such, they are laterally situated in the myosepta, whereas the intermuscular bones are located deeper and more medially. They may be located in the epineural and epipleural positions, posterior and superficial to the axially-attached intermuscular elements, and may also occur in other positions along the length of the myomeric boundaries, in association with each flexure of the superficially multiply-deflected faces of the myotomes.

The superficial and highly organized nature of the elements described herein in the tail of pachycephalosaurids suggest that they are myorhabdoid structures, ossifications otherwise unknown in tetrapods. The trunk musculature of tetrapods is highly modified, although it begins from a myoseptal/myotomal arrangement from which sheets may coalesce over broad expanses of the trunk, obscuring the initial segmental architecture [14]. The tail, however, extends posterior to the region of the body occupied by the coelomic cavity, and has retained a segmental patterning of its musculature that is much more similar to the trunk musculature of fishes. This is expressed as a nested cone-in-cone architecture, that is lost in the trunk of tetrapods [14], with muscle blocks extending by folding and deflecting the segmental boundaries beyond the anterior and posterior extremities of the vertebral pair that they primitively straddle. This pattern is immediately evident in the muscle blocks in the tails of lepidosauromorphs [15]. Segmental boundaries are marked by zig-zag lines of muscle intersection that mark the positions of the myorhabdoi of pachycephalosaurids and represent a halo-like disposition of intermuscular boundaries that lie around the periphery of the tail, extending from the region of the neural spines dorsally to the position of the chevron bones ventrally.

**Figure 7:**
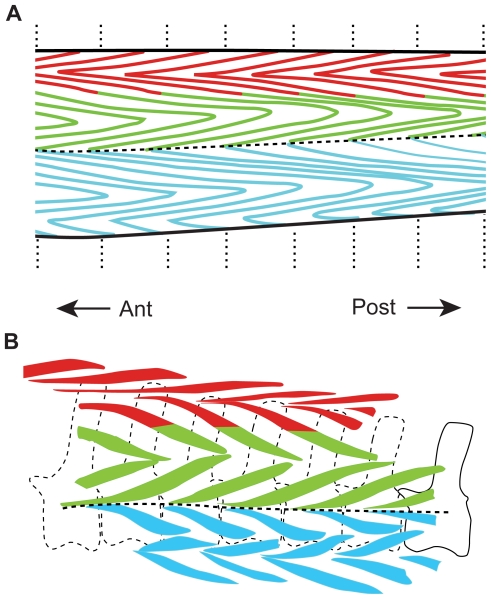
Proposed homologies of the caudal musculature of crocodilians with that of *Homalocephale*. The proposed homologies of (**A**) the caudal musculature of crocodilians (*Gavialis* – Modified from Frey [45]), with (**B**) the six tendon rows of Maryańska and Osmólska [5] in *Homalocephale*. Anterior oriented to the left. Red = *M. spinalis caudae* (*M. tendino-articularis*), Green = *M. longissimus caudae*, Blue = *M. ilio-ischio-caudalis*, Horizontal dashed line = horizontal skeletogenous septum, vertical dashed lines = vertebral boundaries.

A similar pattern of morphology of caudal myomeres and myosepta is seen within extant crocodilians, which are often used as modern analogs for dinosaurs, including recent approaches to the reconstruction of caudal musculature [16]–[19]. They are advocated to be a good model for comparison with dinosaurs given their phylogenetic position [20], [21]. The similarity of the tendinous architecture therefore allows for a hypothesis of homology between the preserved pachycephalosaurid ossified tendons, and the muscles and tendons of extant crocodilians to be advanced. The superficial crocodilian tail musculature is composed of three muscle groups. Employing the nomenclature of Arbour [15] and Persons and Currie [14], the epaxial muscle is characterized by the dorsomedial, anteriorly-directed *M. spinalis caudae* (*M. tendino-articularis*) and the more ventrolateral and posteriorly-oriented *M. longissimus* (Figure 7A). The hypaxial musculature consists of the ventrally placed *M. ilio-ischio-caudalis*. Tendon rows one and two would form the dorsalmost rows, and furnish an anteriorly directed V at their interface (Figure 7B). These ossified tendons of pachycephalosaurs appear to be homologous with the myosepta of the *M. spinalis caudae* (*M. tendino-articularis*). Tendon rows two and three lie ventrolateral to the *M. spinalis caudae* and form an anteriorly directed V at their interface. These tendons appear to be homologous with the location of the myosepta of the *M. longissimus caudae*. The anteriorly-directed V at the ventral margin of row three and the dorsal margin of row four is located at the approximate level of the caudal transverse processes, and as such represent the location of the horizontal skeletogenous septum and the segregation of epaxial and hypaxial musculature. This is similar to the morphology seen in the caudal musculature of extant crocodilians (Figure 7A). Tendon rows four, five and six represent alternating muscle segments of the *M. ilio-ischio-caudalis*.

The exceptionally preserved compsognathid *Scipionyx* preserves multiple soft tissue structures, including what are interpreted as somatic muscle bundles from the base of the tail [22]. These structures are exquisitely preserved, with sacromere level striations attributed to the *M. caudofemoralis* and *M. ilio-ischiocaudalis*. This rare preservation of soft tissue myomeres and myosepta in a non-avian theropod would allow for comparison with the ossified structures preserved in pachycephalosaur tails, but in *Scipionyx* is limited in regional extent so that the architecture of the myosepta cannot be thoroughly documented.

Recent studies have mapped the configuration of myoseptal tendons in craniates [14], [23] permitting further contextualization of the findings of Patterson and Johnson [12]. The gnathostome ancestor possessed a highly-complex myoseptal collagen architecture forming sheets separating the folded myomeric musculature. This arrangement has undergone few changes in the 400 Ma history of vertebrate evolution.

**Figure 8:**
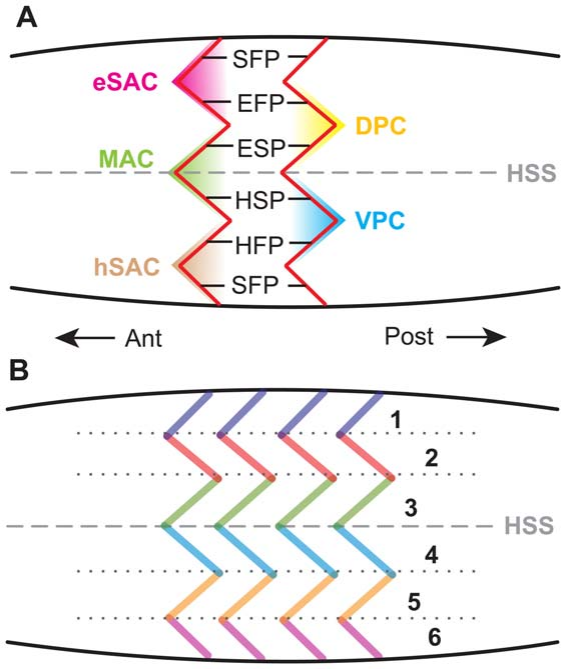
Proposed homologies of the myoseptal segments of teleosts with those of *Homalocephale*. The proposed homologies of the myoseptal segments of Gemballa et al. [25] (**A**) with six tendon rows of Maryańska and Osmólska [5] (**B**).

Gemballa et al. [14], [24] determined that the myospetum is primitively characterized by a single Main Anteriorly-projecting Cone (MAC) (Figure 8A) straddling the horizontal skeletogenous septum, a Dorsal and a Ventral Posteriorly-projecting Cone (DPC and VPC) immediately dorsal and ventral to the MAC respectively, and, often, epaxial and hypaxial Secondary Anteriorly-projecting Cones (eSAC and hSAC) (Figure 8A), all of which nest within the cones of adjacent myosepta (Figure 8A). The lateral profile reveals a zig-zag arrangement, with sections of the zig-zag boundary receiving names — the inner (those adjacent to the Horizontal Skeletogenous Septum — HSS) dorsal and ventral segments being termed the Epaxial Sloping Part (ESP) and Hypaxial Sloping Part (HSP), the middle segments being termed the Epaxial Flanking Part (EFP) and Hypaxial Flanking Part (HFP), and the outer segments being termed the Secondary Flanking Parts (SFP) (Figure 8A). Myorhabdoid tendons are located within both the Epaxial and Hypaxial Flanking Parts, and lateral tendons are situated within both the Epaxial and Hypaxial Sloping Parts. An epineural tendon and an epipleural tendon are located within the Epaxial Sloping Part and the Hypaxial Sloping Part, respectively. These latter structures lie deeper within the muscular cone, are absent from the trunk region of tetrapods [14], and only the myorhabdoid tendons are exposed on the surface. These patterns of disposition segregate the myorhabdoid bones from the intermuscular bones as described by Patterson and Johnson [12].

The actinopterygian epicentral intermuscular bone [12] has a medial to lateral course and may have a forked lateral extremity, with the apex of the fork oriented anteriorly. The lateral tendons may coossify with the distal end of the epicentral intermuscular bone to give rise to a Y-shaped element. This may be the source of coalescence of the type 2 elements, as depicted in Figure 4, although the details of myoseptal structure in the tail of tetrapods has not been documented to the same degree as that for the trunk [14]. If this fails to occur, then rod-like individual myorhabdoi would flank the region of the Horizontal Skeletogenous Septum dorsally and ventrally.

In light of the reconstructed architecture of the ossified caudal complex in pachycephalosaurids, we suggest that the ossified caudal elements are thus not fundamentally *de novo* structures, but rather are homologous with the myoseptal tendons of non-tetrapod craniates, manifested as ossified units termed myorhabdoi. The ossification of these structures is homoplasious with the condition manifested in teleosts [9]. Thus, it is not the positional morphology of these structures that is unique, but rather their ossification, and therefore their fossilization. The serially homologous “V” shaped structures in the tail of pachycephalosaurids are interpreted here as the nested cone in cone structure of the myosepta at, or very near, the interface with the integument. The ESP and HSP of Gemballa et al. [25] (Figure 7A) are homologous with rows three and four, respectively, of Maryańska and Osmólska [5] (Figure 8B). The myosepta associated with the EFP and EFP are homologous with rows two and five (Figure 8A, 8B), and the myosepta of the SFP (epaxial) and SFP (hypaxial) are homologous with rows one and six (Figure 8A, 8B).

**Figure 9:**
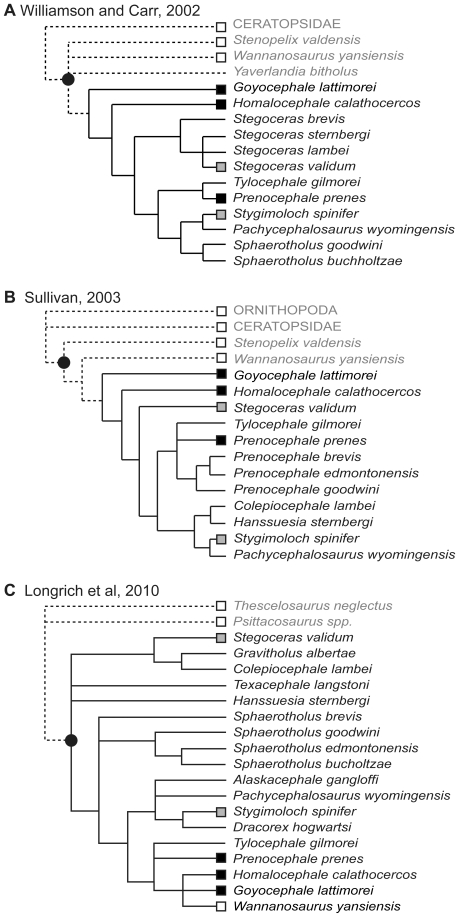
Occurrence of myorhabdoid ossifications in pachycephalosaurid taxa. Occurrence of myorhabdoid ossifications in pachycephalosaurid taxa based upon three recent hypotheses of pachycephalosaur relationships: **A**) Williamson and Carr [46]; **B**) Sullivan [47]; **C**) Longrich et al. [48]. Solid circle demarcates Pachycephalosauridae. Squares indicate taxa with significant postcranial material; white = no evidence of myorhabdoid ossifications, black = articulated myorhabdoid ossifications, grey = disarticulated myorhabdoid ossifications. Solid lines indicate inferred presence of myorhabdoid ossifications based on topology. Taxonomic nomenclature follows that of each original publication.

All pachycephalosaurid postcranial skeletons examined herein preserve either articulated or associated superficial caudal elements (Figure 9). This sample encompasses nearly all taxa (and most specimens) for which material other than the cranium is preserved (Figure 9). This suggests that the presence of these structures is ubiquitous within this clade, and likely occurred in many taxa currently known only from cranial material (Figure 9). No other dinosaurian clade exhibits such structures, suggesting that their ossification is a synapomorphy of the Pachycephalosauria.

Pachycephalosaurid superficial ossified caudal elements are distinct in both their structure and positional location from the ossified tendons reported for other ornithischians [11], [26]. Within the Ornithopoda, relatively small taxa such as *Tenontosaurus*, *Parksosaurus*, *Oryctodromeus*, *Thescelosaurus* exhibit ossified tendons as bundles composed of straight rod-like elements either epaxially, or both epaxially and hypaxially, in the caudal region [11], [27]–[30].

The lattice of ossified tendons that occurs in iguanodontian dinosaurs [11], [26], [31]–[33] consist of epaxial structures only, and reside deep relative to the trunk musculature, along the spinous processes of the thoracic, sacral, and caudal vertebrae. As such, these structures are more akin to the derivatives of the ligaments associated with the intermuscular epaxial bones of osteichthians and have been hypothesized to be homologous to the *M. transversosinalis* in crocodilians and the *M. longus colli dorsalis*, *pars thoracics* of birds [26]. Kafuku [34] identified anterior oblique and posterior oblique tendons that are associated with the vertebrae and that may span from three to eight vertebral segments. The anterior oblique tendons ossify in some teleost fishes [12]. Other similar tendons below the horizontal skeletogenous septum are also expressed in teleosts, in series with or including epineural and epipleural bones [12].

Ceratopsids display epaxial tendons with a similar morphology to those of iguanodontians. As for iguanodontians, these structures are arranged in three distinct layers that are closely associated with the neural spines, and are not superficial [35]. These structures are thought to be homologous with those seen in iguanodontians [35].

Ankylosaurids have a series of ossified tendons in their tails lateral to the vertebral column and that extend along the handle of the tail [11], [36]. These elements can be divided into a deep and shorter imbricated series, and a more superficial, longer and parallel to braided series [18]. These are thought to be associated with muscles used to wield the caudal club.

Within Theropoda, specifically Tetanurae, stiffening of the tail is often accomplished by zygapophyseal extensions [11], [37], [38]. This is perhaps most extreme in *Deinonychus*, in which such extended zygapophyses may span up to twelve vertebral segments [37]. Intriguingly, Ostrom originally suggested these structures might have originated as ossification of caudal muscle tissue with later association with the zygapophyses [37]. This view, although not currently supported [11], [38], is similar to our interpretation of the pattern of ossification within pachycephalosaurs.

The morphologies of all other dinosaurian ossified tendons contrast markedly with the superficial ossifications found in the tails of pachycephalosaurs. In the latter situation the elements are relatively short in comparison, are fusiform and are disposed in a zig-zag pattern that is restricted to the periphery of the tail (in a location approximating the interface of the caudal musculature and the integument). One potential exception to this restricted occurrence of these elements lies in the tail of a specimen of *Hypsilophodon*. This specimen (NHMUK R196) preserves somewhat superficial elements that are roughly V-shaped, and that are disposed in potentially serially homologous rows. Although these elements are distinctive when compared to those of pachycephalosaurids, they do show general similarities. Due to incomplete preservation, full discussion of their anatomy and potential homology is not currently possible.

In addition to being morphologically distinct, the pachycephalosaurid elements are also histologically distinctive, incorporating relatively more bone than do the ossified tendons of all other dinosaurs. The tendons of *Stygimoloch* have a loosely packed core consisting of Haversian bone and longitudinally-oriented collagen fibers, and an outer region of tightly packed fibrolamellar tissue [11]. This histological organization exhibits vascularity in the outer region that alternates between longitudinal and radial, and has been described as being “plywood-like” in pterosaur bones [39]. This arrangement has been advocated to be an adaptation for effectively resisting torsional loading in birds [40]. This is consistent with the cross-fiber texture of the horizontal septum system of gnathostomes [25], providing additional structural evidence for the myoseptal tendon affinity of the superficial caudal ossifications of pachycephalosaurids.

### Palaeobiological implications

Ossification of the myoseptal tendons has previously only been reported in teleosts [12], [25]. Within these taxa, however, the myoseptal ossifications are small relative to the size of the myomeres and myosepta. The ossified structures seen in pachycephalosaurids are, in comparison, massive, occupying almost the entire length of the myoseptum, and are thick, being nearly as thick anteroposteriorly as the adjacent myomeres, suggestive that the periphery of the tail was composed of nearly as much bone as muscle tissue. Such extreme morphology invites discussion of its functional consequences.

The extent of the myorhabdoi and their relationship to the underlying vertebrae indicate a highly folded nature of the myomeres that extend across as many as five or six vertebral segments. The myorhabdoi provide rigid lateral sites of insertion for the segmental tail muscles (fiber direction cannot be determined) and would add significant rigidity to the periphery of the tail. Such ossified intersections may have endowed the tail with particular mechanical properties, which would be dependent upon the direction of loading.

The interspersion of myorhabdoi along its length would likely increase the potential rigidity of the tail during myomeric muscle contraction. If buttressed against the substrate such a rigidified tail could enhance its ability to participate in a “tripodal prop”, a function suggested in the original description [5]. This type of supportive involvement may also be consistent with the pattern of placement and relationship of the epaxial and hypaxial myorhabdoi. The epaxial components are spaced more widely apart than the hypaxial ones (Figure 2C, 2D), and have greater angles between the long axis of the expanded middle portions of the elements. If used as a prop, the hypaxial region of the tail would be subjected to tensile loading, and the epaxial part to compressive loading, especially in the region between the base of the tail and the point at which the tail made contact with the substratum. If the tripodal support contributed by the tail was subjected to lateral loading, torsional forces would also be applied to the tail. The “plywood-like” histological configuration of the tissue layers in the myorhabdoi [11] would be appropriately disposed to resist torsional loading [40]. The hypothesized function of the tail as part of a tripodal prop mechanism in pachycephalosaurids is concordant with the presence of robust caudal myorhabdoi. This association is not sufficient to confirm such a function, but it is consistent with such a role.

The suggestion that the ossified myosepta may have acted in a defensive manner, such as a cuirass, related to agonistic flank butting [10] also deserves comment, given the additional knowledge of its architecture. Although the ossified myosepta are densely packed and form a nearly continuous halo of twelve elements around the circumference of a tail's cross-section, significant gaps between the adjacent segmental complexes do exist. Comparisons with other extant and extinct amniotes for which superficial armor is better known (e.g. xenarthrans, ankylosaurs and crocodilians) show either a condition of completely fused osteoderms, or smaller spacing between isolated elements. Additionally, osteoderms in these taxa are placed more superficially, within the dermis, rather than within the periphery of the muscle tissue. The ossified myosepta of pachycephalosaurs are therefore not consistent with the pattern of cuirass armor in other amniotes, and would likely not have functioned in the same manner.

The ossified myosepta described herein are not the only unique morphology the tails of Pachycephalosauria. Multiple taxa preserve anterior caudal vertebrae that articulate with greatly elongated, and highly bowed, caudal ribs [3], [5]. Additionally, *Homalocephale* (MPC-D 100/1201) preserves an anteriormost caudal rib that is thickened distally and makes sutural contact with the ilium [5]. The co-occurrence of these two morphological features in the same anatomical region, and their common association with caudal musculature, suggests they may have a common or correlated function. Unfortunately, although both are preserved in multiple specimens, none preserve an overlap of the elongate caudal ribs and ossified myosepta (or are well-preserved enough to establish a lack of overlap). As such, it is unclear whether these two features are integrated, but discovery of new, more complete anatomical representation of this area will permit further evaluation.

At this point it remains unclear as to whether there is a functional correlation between the massive and ubiquitous ossified caudal myorhabdoi, and the thickened, and often domed skull roofs characteristic of pachycephalosaurs. The large size, consistency of development, regularity of disposition, unusual location and anatomical derivation of the superficial skeletal structures in the tail of pachycephalosaurids do collectively suggest that they were structures of functional importance. The potential for a functional association of these features requires further investigation. The myorhabdoid ossifications are not homologous with the ossified tendons of other ornithischians [11], but do constitute a feature seemingly synapomorphic for this clade [41], [42]. They represent novel modifications potentially associated with stiffening of the tail. If the frequently advocated antagonistic behavior of the use of the thickened skull roof as a weapon during intraspecific combat [43], [44] occurred, then a tripodal stance, with the tail as one of the supports, would likely have resulted in torsional loading as blows were both delivered and received. In this context it is possible that the myorhabdoi, with their particular histological structure [8], were involved in the resistance of compressive, tensile and torsional loading, and that their presence may be associated with this particular pattern of intraspecific behavior.

### Conclusion

Soft tissue correlates of the previously described caudal tendinous “basket” of pachycephalosaurs are homologous to those of the ossified myorhabdoi in the myosepta of many extant teleost fish, but the ossification of these structures is homoplasious. Recognition of this convergent feature not only marks the first recorded occurrence of such structures in a tetrapod, but also the most extensive and robust expression of these ossifications in any animal group. These structures also allow for a reconstruction of the caudal musculature of pachycephalosaurs, assisted by comparison with modern relatives such as crocodilians. It is unclear at this point whether there are functional correlates between the massive and ubiquitous ossified caudal myorhabdoi, and the thickened, and often domed, skull roofs characteristic of pachycephalosaurs, but such a correlation is not unfeasible.
